# Trip killers: Addressing a critical knowledge gap in psychedelic research

**DOI:** 10.1177/02698811261431056

**Published:** 2026-03-23

**Authors:** Brian O’Mahony, Colm Harrington, Andrew Harkin, Níall Lally

**Affiliations:** 1 University College Dublin, Ireland; 2 Department of Psychiatry, Galway University Hospital, Republic of Ireland; 3 Trinity College Institute of Neuroscience, Trinity College Dublin, Ireland; 4 Children’s Health Ireland, Crumlin, Dublin, Ireland

**Keywords:** bad trips, psychedelic, LSD, psilocybin, antipsychotics, 5-HT2A

## Abstract

Psychedelic drugs are increasingly under investigation as potential therapeutic agents for mental health conditions and are being increasingly used recreationally. Psychedelic use may result in an episode of intense psychological distress, commonly referred to as a “bad trip.” Bad trips represent a potentially volatile, erratic, and dangerous situation, which may, in extreme cases, require presentation to accident and emergency departments and psychiatric hospital admission. Managing such cases requires careful consideration, with priority given to non-pharmacological strategies. When these measures prove insufficient, an alternative approach may be necessary, one that can effectively attenuate or terminate the psychedelic state and restore psychological stability. Despite clinical relevance, there is no systematic evaluation of pharmacological interventions to terminate such experiences. This review identifies and critically appraises candidate medications with potential utility as abortive agents, including serotonin antagonists, drugs for psychosis, and select drugs for anxiety and depression. We review these agents, their mechanisms of action, pharmacokinetics, safety profiles, and applicability in acute care settings. Binding strength at the molecular level, potency to functionally block receptor-mediated effects, and lack of side effects are key considerations. We conclude by proposing a provisional framework for the pharmacologic management of adverse psychedelic experiences and highlight key priorities for future research.

## Introduction

Psychedelic compounds are receiving increasing attention in clinical research as potential therapeutic agents, particularly in the treatment of complex and refractory mental health conditions (Andersen et al., 2021). For example, psilocybin, the psychoactive chemical found in “magic mushrooms,” has shown promise in alleviating treatment-resistant major depression (Carhart-Harris et al., 2021; Goodwin et al., 2022) and anxiety disorders (Griffiths et al., 2016). Classical psychedelic substances such as lysergic acid diethylamide (LSD) and psilocybin produce profound alterations in consciousness, perception, and affect, most notably via intensified visual and auditory phenomena, ego dissolution, and altered self-referential processing (Johnson et al., 2008). These phenomenological effects are hypothesized to mediate therapeutic benefit by facilitating psychological insight, catharsis, and a transient increase in neural and cognitive flexibility (Carhart-Harris and Friston, 2019; Roseman et al., 2018), although direct evidence is lacking (Barbut Siva et al., 2024; Goodwin et al., 2023; Olson, 2020).

These same properties can also lead to intensely distressing experiences, commonly referred to as “bad trips,” a term that emerged in the 1960s subculture, where psychedelics were thought to enable a voyage or “trip” into the unconscious. Bad trips have been long described in literature (Bunce, 1979) and can entail severe anxiety, paranoia, and perceptual disturbance (Kopra et al., 2022a, 2022b; Simon et al., 2024). These distressing effects can be experienced in a variety of modalities, such as sensory, somatic, psychological, and metaphysical (Johnson et al., 2008). Such bad trips are more likely when the user’s mindset (“set”) or environment (“setting”) is suboptimal (Carhart-Harris et al., 2018). Psychedelic use is increasing, with data suggesting a near doubling of last-year usage in the United States from 2015 (4.7 million users) to 2022 (8.5 million users; (Center for Behavioral Health Statistics and Quality, 2016, 2023). As psychedelic use increases, a greater number of individuals are using these substances in uncontrolled environments, such as community gatherings, underground retreats, or independently at home (Kettner et al., 2021). Presentations with significant distress post-psychedelic use have become increasingly common in emergency departments (Simon et al., 2024), meaning this phenomenon is relevant to clinicians. Most bad trips will settle without pharmacological management, and almost all will self-resolve within 24 hours (Kopra et al., 2022a, 2022b). However, the severity of a portion of bad trips may reach the point of requiring pharmacological intervention, and the lack of consensus or guidelines on the subject means that a wide range of treatments are utilized (Simon et al., 2024). While clinical trials have “rescue medication” available, there is no consensus, and the rigorous attention to set and setting mean these are rarely used (Davis et al., 2021a; Goodwin et al., 2022; Von Rotz et al., 2023). However, non-clinical and recreational use are unlikely to provide this level of safety-netting, and it is in this context that we write this review.

In this article, we review the theoretical basis, empirical evidence, and safety profiles of pharmacological agents that may be used to terminate or attenuate a psychedelic experience. We focus on classic serotonergic psychedelics such as psilocybin and LSD, given their prominence in both clinical trials and recreational contexts. This article also applies to terminating psychedelic experiences brought on by use of N, N-Dimethyltryptamine (DMT) and ayahuasca, whose primary psychoactive component is DMT, as well as mescaline. It is less likely to be useful in terminating experiences secondary to non-classical psychedelics, such as 3,4-Methylenedioxymethamphetamine (MDMA), whose primary mechanisms of action do not involve activation of the Serotonin 2A (5-HT_2A_) receptor. We also examine non-pharmacological interventions used in clinical and informal settings and explore the ethical considerations associated with interrupting a potentially therapeutic, albeit challenging, psychological experience.

## Non-pharmacological management of bad trips

Pharmacological interventions should be considered only when the bad trip is judged to be unresponsive or unsuitable to non-pharmacological interventions, for example, when there is a high chance of the person or others being seriously harmed. Even when a patient is judged to be in extreme distress, this experience may hold therapeutic value (Johnson et al., 2008; Wood et al., 2024). Psychological containment, a process of emotional space holding by another individual or clinician, may allow for meaningful processing of a difficult psychedelic experience to occur within a safe framework. This approach emphasizes working with and through, rather than avoiding, emotional difficulty and can be achieved by clinicians remaining calm, non-judgmental, and emotionally regulated (Johnson et al., 2008; Noorani, 2021). The processing of difficult psychedelic experiences has been shown to lead to significant psychological breakthroughs in depressive symptoms (Griffiths et al., 2016; Roseman et al., 2018) and self-understanding (Haijen et al., 2018) and can influence whether a crisis escalates or is transformed into an opportunity for healing (Phelps, 2017; Richards, 2015).

The concept of set and setting, which includes mindset, environment, and interpersonal context, plays a crucial role in determining the trajectory and lasting outcome of psychedelic experience (Hartogsohn, 2016; Zinberg, 1984). Achieving an optimal setting is likely to be difficult in the medical context, particularly in emergency departments designed to manage physical injury and psychiatric crises involving imminent risk, rather than supporting individuals in altered states of consciousness. The person should be placed in a calming, low-stimulus environment to complement the interpersonal support previously outlined. Once the acute distress of a bad trip has subsided, integration becomes a vital component of care. Integration occurs in the days to weeks following the psychedelic experience, and refers to the process of making sense of the psychedelic experience, applying insights to everyday life, and consolidating emotional or existential learning (Carhart-Harris et al., 2018; Watts et al., 2017). Without support, individuals may remain confused or emotionally unsettled after intense experiences, which can be avoided with structured debriefing and peer support (Modlin et al., 2025; Pleet et al., 2023). As psychedelic use becomes more widespread, health systems must be prepared to recognize when patients are experiencing a bad trip and to provide supportive non-pharmacological and integrative care to reduce potential harm. In some cases, however, these approaches may be insufficient or unsuitable, and inadequate management can result in lasting psychological consequences (Evans et al., 2025). Thus, clinicians should be aware of pharmacological options for bad trips and their mechanisms of action, side effect profiles, and what is likely to be efficacious and what is not.

## Pharmacology of psychedelics

Although classical psychedelics have a diverse receptor binding profile, it is widely accepted that the induction of the psychedelic state is mediated through agonism of the 5-HT_2A_ receptor (Becker et al., 2023; Holze et al., 2024a; Vollenweider et al., 1998), which itself couples to multiple G proteins and signaling pathways and displays biased agonism (functional selectivity; Martí-Solan et al. 2015). This means different ligands (e.g., LSD, psilocybin) can stabilize different receptor conformations and activate different downstream effects. As such, a psychedelic’s or medication’s effect is more complex than binary agonism or antagonism of the receptor. The degree to which a drug binds to the 5-HT_2A_ receptor, the binding affinity (Ki), and the time spent occupying that receptor, the *K*_off_ or dissociation rate, likely correlate with the clinical potency and thus the amount of drug needed to induce the psychedelic effect. For example, LSD and psilocybin have high affinity for the 5-HT_2A_ receptor (see Table 1 for Ki values, psychedelic half-lives). Some psychedelics also recruit other processes such as beta arrestins, intracellular proteins that act as a signaling scaffold and influence signal sensitisation and duration (McClure-Begley and Roth, 2022). For example, LSD strongly recruits beta arrestins, (Rodriguiz et al., 2021) but DMT does not (Blough et al., 2014). Additionally, preclinical evidence suggests that LSD’s psychedelic drug-like actions appear to require beta-arrestin 2, and not beta arrestin 1 (Rodriguiz et al., 2021). Nonetheless, blockade of 5-HT_2A_ receptor should inhibit psychedelics from activating the receptor and thus prevent the induction of a psychedelic state. Duration of action is more fully explained by the differences in elimination half-lives rather than by differences in receptor interaction (Table 1).

**Table 1.** Pharmacological properties of select psychedelics.

Psychedelic drugs	5-HT_2A_ binding affinity (Ki)	Additional receptor profile	Time to peak effect (hours)	Half-life (hours)	Enzymatic parameters and potential interactions	Common side effects
3,4-Methylenedioxymethamphetamine (MDMA; Green et al., 2003; Kalant, 2001; Sarparast et al., 2022; Simmler and Liechti, 2018)	~7800 nM (low direct affinity for hallucinogenic effects). MDMA’s effects are primarily mediated by its action as a monoamine releaser	Primarily a potent serotonin (5-HT), norepinephrine (NE), and dopamine (DA) releasing agent, and a reuptake inhibitor	1.5-2.5 (oral) [variable]	6–8 hours. Subjective effects lasting 3–6 hours	Primarily metabolized by CYP2D6 and CYP3A4. Many drug interactions due to CYP2D6/3A4 inhibition/induction	Increased heart rate and blood pressure, hyperthermia (overheating), jaw clenching (bruxism), nausea, loss of appetite, sweating, dehydration, hyponatremia (low sodium. Risk of serotonin syndrome
4-bromo-2,5-dimethoxyphenethylamine (2-CB; Rickli et al., 2015; Thomann et al., 2025; Villalobos et al., 2004)	Low efficacy partial agonist/functional antagonist. Ki 5-11 nM (Papaseit et al., 2018)	Partial agonist of 5-HT_2B_, and 5-HT_2C_	1–2 (oral), peak subjective effects at 2 hours	1–1.5 hours, with subjective effects lasting 4–8 hours	Primarily metabolized by MAO-A and MAO-B enzymes, also by CYP 2D6	Nausea, vomiting, “jitters” (body tremors), muscle spasms
Ayahuasca (Carbonaro and Gatch, 2016; Callaway et al., 1999; Rickli et al., 2016; Ruffell et al., 2020, 2023)	Primary psychoactive component is DMT. DMT’s 5-HT_2A_ Ki is ~75 nM (for DMT itself)	Contains harmala alkaloids, which are reversible MAO-A inhibitors (RIMAs). This inhibition prevents the breakdown of orally ingested DMT, allowing it to become orally active. Also interacts with other serotonin receptors (5-HT_1A_, 5-HT_1B_, 5-HT_1D_, 5-HT_2C_), and to a lesser extent, dopamine and adrenergic receptors.	1–2	DMT: 4–19 minutes when orally active due to MAOI. Harmine 1.9–2.0 hours, Harmaline 4–8 hours. Subjective effects lasting 4–8 hours	MAO-A inhibition by harmala alkaloids leads to significant drug–drug interactions with substances that affect serotonin, such as SSRIs, SNRIs, MDMA, amphetamines, and certain foods (tyramine-containing). Can lead to serotonin syndrome or hypertensive crisis	Nausea, vomiting, diarrhea (often considered part of the “purging” process in ceremonial use, termed “la purga”), increased heart rate and blood pressure
Lysergic acid diethylamide (LSD; Ali et al., 2025; De Gregorio et al., 2016a, 2016b; Dolder et al., 2017; Holze et al., 2024a; Rickli et al., 2016)	~1.5–5 nM (high affinity)	Partial agonist at 5-HT_1A_, 5-HT_1B_, 5-HT_1D_, 5-HT_2B_, 5-HT_2C_, 5-HT_5A_, 5-HT_6_, 5-HT_7_), dopamine receptors (D_1_, D_2_), and adrenergic receptors (α1, α2)	1–2.5 (oral)	3–5 hours (average 3.6 hours). Displays prolonged residence time at 5-HT_2A_, leading to sustained activation and subjective effects (6–12 hours) after the drug has been cleared	Primarily metabolized by CYP2D6 and to a lesser extent by CYP2C9, CYP2E1. Potential for drug interactions with CYP2D6 inhibitors or inducers	Dilated pupils, increased heart rate and blood pressure, nausea, dizziness, confusion, altered perception of time and space, hallucinogen persisting perception disorder (HPPD)
Mescaline (Halberstadt et al., 2013; Kolaczynska et al., 2022; Rickli et al., 2016; Uthaug et al., 2022)	Partial agonist, Ki ~10,000 nM (comparatively low affinity, but high efficacy)	Also interacts with 5-HT_2B_, 5-HT_2C_, adrenergic, and dopamine receptors, but with lower affinity for non-serotonergic targets	1–3 (oral)	2.5–6 hours, although subjective effects last for 8–12 hours	Primarily metabolized by oxidative deamination via MAO enzymes, then followed by aldehyde dehydrogenase to form trimethoxyphenylacetic acid	Nausea, vomiting, diarrhea (viewed as part of a ceremonial “cleanse” or “purge”), increased heart rate and blood pressure, sweating, chills, muscle tremors
N, N-Dimethyltryptamine (DMT; Carbonaro and Gatch, 2016; Rickli et al., 2016; Van Der Heijden et al., 2025)	~75 nM (moderate affinity)	Primarily a full agonist at 5-HT_2A_ receptors. Also binds to 5-HT_1A_, 5-HT_1B_, 5-HT_1D_, 5-HT_2C_, 5-HT_7_, and sigma-1 receptors	Inhaled/IV: 0.067–0.33 (4–19 minutes)	Inhaled/IV: ~0.1–0.25 (4–19 minutes). Oral DMT alone is not active due to rapid breakdown by MAO. Subjective effects lasting 15–30 minutes	Rapidly metabolized by monoamine oxidase (MAO) enzymes (primarily MAO-A) in the gut and liver. Co-ingestion with MAO inhibitors (e.g., in ayahuasca) is necessary for oral activity	Increased heart rate and blood pressure, nausea, anxiety
Psilocybin (prodrug for Psilocin; Madsen et al., 2019; Meshkat et al., 2025; Sakashita et al., 2015)	Psilocin (active metabolite) is a partial agonist, ki ~10–20 nM. Psilocybin itself has negligible affinity for serotoninergic receptors	Also interacts with 5-HT_1A_, 5-HT_1B_, 5-HT_1D_, 5-HT_2C_, 5-HT_6_, and 5-HT_7_ receptors	Psilocin peak effect: 0.5–1.5 (oral psilocybin)	Psilocin: 2–4 hours. Psilocybin: very short, rapidly converted to psilocin. Subjective effects lasting 3–6 hours	Psilocybin, upon oral ingestion, is dephosphorylated to psilocin. Psilocin is then metabolized by glucuronidation and by CYP450 enzymes (*CYP2D6*, *CYP3A4*, *CYP2C9*, *CYP2C19*)	Nausea, stomach discomfort, muscle weakness, yawning, increased heart rate, and blood pressure

In addition to the 5-HT_2A_ receptor, classic psychedelics also act on other serotonergic receptor sites (Blair et al., 2000; McKenna et al., 1990). Of particular note for this article is the role of the 5-HT_1A_ receptor, one of the main inhibitory receptors of the serotonergic system (Albert and Vahid-Ansari, 2019). The 5-HT_1A_ receptor is found post-synaptically throughout the cortex but is also found somatodentrically in the raphe nuclei, where it acts as autoreceptor to inhibit the firing of serotonergic neurons and subsequent 5-HT release throughout the forebrain (Barnes and Sharp, 1999). Intriguingly, pre-treatment with buspirone, a 5-HT_1A_ full presynaptic agonist and partial agonist post-synaptically with limited activity at the 5-HT_2A_ receptor, (Hjorth and Carlsson, 1982) reduced the psychedelic effects of psilocybin in humans (Pokorny et al., 2016). However, it is currently unknown how 5-HT_1A_ receptor agonists impact psychedelic experiences when given during a psychedelic experience. One possibility is that modulating the autoreceptor activity in the raphe nuclei may be insufficient to terminate an already occurring postsynaptic-mediated experience.

Other receptors of note include glutamatergic receptors, given psychedelics indirectly increase glutamate in the prefrontal cortex via the 5-HT_2A_ receptor (Petrušková et al., 2025). Glutamatergic NMDA receptor involvement is crucial to induce the synaptic plasticity thought to play a role in the beneficial effects of psychedelics in mental disorders (Weiss et al., 2025). Other receptors may be relevant depending on the psychedelic, for example, dopamine 2 receptors at high doses of LSD; sigma-1 receptor in DMT and related tryptamines; and trace amine-associated receptor 1 in phenylethylamines (De Gregorio et al., 2016a; Fontanilla et al., 2009; Seeman et al., 2005; Xie & Miller, 2008). However, these receptors have secondary relevance to the 5-HT_2A_ receptor in inducing a psychedelic experience. Thus, the focus of this review hereafter primarily focuses on 5-HT_2A_ receptor antagonism as the most likely candidate mechanism of action for termination of a bad trip.

## Pharmacological management of challenging psychedelic experiences

### “The perfect trip killer”

Given the well-established role of 5-HT_2A_ receptor agonism in mediating psychedelic effects, one might assume that antagonism at this site would provide a straightforward solution for aborting adverse experiences. If the antagonist possesses a lower binding affinity relative to the agonist, or if the agonist concentration at the receptor site remains significantly elevated, achieving complete receptor blockade becomes dynamically challenging, requiring higher doses in the case of competitive antagonists, while non-competitive antagonists are less likely to surmount the effects of the agonist. To achieve rapid cessation of the bad trip, the medication would need to be rapidly delivered to the receptor, necessitating either a drug with rapid time to peak plasma for oral drugs, or for use intravenous, intranasal or rapid-acting intramuscular administration.

A further consideration is for the antagonist potency, i.e., how much of it is needed to produce the functional effect. Potency for antagonists is usually measured as IC_50_ derived from binding and functional assays and depends on not just affinity but also the drug’s pharmacokinetics at the relevant site. With respect to LSD, affinity informs as to whether an antagonist can compete at the 5-HT_2A_ receptor binding site. If the antagonist’s affinity is weaker than LSD it will not block LSD binding to the 5-HT_2A_ receptor effectively unless given at high doses. Potency tells us how much antagonist (dose and available concentration at the target site) is required in the brain to actually overcome LSD’s effects. Even a high affinity antagonist may be low in potency in vivo if it has poor brain penetration or rapid clearance. A high affinity, high potency 5HT_2A_ receptor antagonist that can achieve high cortical 5-HT_2A_ receptor occupancy (80%–90%) at the time LSD is active would therefore be required.

Peak-plasma time and half-life of the trip killer are also important considerations. If the agonist remains bound to the receptor for a prolonged time (i.e., has slow dissociation kinetics), which in the case of LSD is mediated by the folding of an extracellular “lid” over the binding pocket (Kim et al., 2020), even a high-affinity antagonist may need sustained high concentrations or extended time to achieve effective receptor occupancy. This would thereby delay functional antagonism and could prolong psychedelic effects. Failure to account for this may lead only to temporary relief from the bad trip, depending on what psychedelic has been taken and when during the trip the person presents and seeks support. The ideal trip-killer would possess a rapid onset of action (ideally within 5–15 minutes), be easy to administer (e.g., oral, sublingual routes, intranasal and intramuscular), be available in emergency department or hospital settings, be affordable, and act through a selective mechanism, most plausibly via 5-HT_2A_ receptor antagonism and have no or limited negative side effects. Additionally, the agent should have a half-life of 4–6 hours; sufficient to outlast peak drug effects without lingering side effects and must have a benign physiological profile, avoiding complications such as respiratory depression, cardiovascular instability, or prolonged sedation. Sedating compounds may undermine therapeutic engagement, obscure residual processing, or introduce new risks in unsupervised environments.

In sum, the ideal abortive compound would be potent, fast-acting, easily administered, selective in its mechanism, and physiologically safe. The development or identification of such an agent represents a critical step in the responsible clinical use of psychedelics.

### 5-HT_2A_ antagonists

Table 2 shows characteristics of select 5-HT_2A_ receptor antagonists. As discussed, blockade of the 5-HT_2A_ receptor offers a mechanistically plausible route to terminating psychedelic experiences. Ketanserin, one of the most studied candidates, has long been known to attenuate psychedelic effects. Ketanserin pretreatment has been shown to prevent the acute effects of LSD (Holze et al., 2021; Preller et al., 2017), psilocybin (Carter et al., 2005; Quednow et al., 2012; Vollenweider et al., 1998) and mescaline (Klaiber et al., 2024) and to reverse the response to LSD (Becker et al., 2023). Ketanserin (40 mg) given 1 hour after LSD (100 µg) in a randomized, double-blind, placebo-controlled crossover trial significantly shortened and attenuated LSD’s acute effects from 8.5 to approximately 3.5 hours without changing the pharmacokinetics of LSD (Becker et al., 2023). This is the clearest human demonstration of true reversal after the psychedelic is underway due to pharmacodynamic interaction. Oral doses of ketanserin result in dose-dependent 5-HT_2A_ receptor blockade (half-maximal occupancy at a moderate dose of 10 mg), making it a practical candidate as a trip killer (Holze et al., 2024a). However, ketanserin, though well-studied in both experimental and cardiovascular contexts, is not widely approved outside of select European and Asian markets and is unavailable in key regions such as the United States and the United Kingdom. Moreover, it is known to cause hypotension and may not be suitable for some individuals.

**Table 2.** Pharmacological properties of select 5-HT_2A_ antagonists/inverse agonists.

Drug	Primary use	5-HT_2A_ binding affinity (Ki)	Additional receptor profile	Time to peak plasma level	Half-life	Advantages/disadvantages
Ketanserin	Hypertension, also studied for chronic pain	2–8 nM (Bryant et al., 1996; Holze et al., 2024a, 2024b; Janowsky et al., 2014)	α_-1_-adrenergic antagonist, 5-HT_2C_ antagonist, H_1_ antagonist (Janowsky et al., 2014)	2–3 hours	Effective half-life: 2–3.5 hours (Becker et al., 2023). Terminal half-life: 14–17 hours (Heykants et al 1986; Persson et al., 1991)	Widely used in research to attenuate psychedelics (Carter et al., 2005; Holze et al., 2024a; Preller et al., 2017). Not widely available in the United States or United Kingdom for routine clinical use, primarily available in some European countries. May cause dizziness, hypotension (especially orthostatic), dry mouth, nausea, edema
Pimavanserin	Psychosis associated with Parkinson’s disease, also approved for dementia-related psychosis in some regions (e.g., United States)	0.1–1 nM (very high affinity; Gardell et al., 2007). Inverse agonism in frontal cortex (Muneta-Arrate et al., 2020)	Highly selective for 5-HT_2A_ and 5-HT_2C_ receptors (inverse agonist/antagonist), minimal affinity for D_2_ or other dopamine receptors (Gardell et al., 2007; Davis et al., 2021b)	2–6 hours (Abbas and Roth, 2008)	57 hours (Davis et al., 2021b)	Approved for clinical use. Risk of QT prolongation. Long half-life. Higher cost. May cause nausea, peripheral edema, confusion, QT prolongation, constipation (Davis et al., 2021b)
Pirenperone	Investigational agent, research tool (selective 5-HT_2A_ antagonist)	0.1–0.2 nM (very high affinity; Kennis et al., 1986)	Highly selective for 5-HT_2A_ receptors; minimal affinity for other receptors (e.g., D_2_, H_1_, α_-1_-adrenergic, muscarinic) at clinically relevant doses (Kennis et al., 1986)	N/A	4–6 hours (Janssen Pharmaceutica v. Mylan Pharmaceuticals, 2007)	High selectivity for 5-HT_2A_ means it has minimal off-target effects. Not clinically approved or widely available for therapeutic use; efficacy in human conditions not fully established. Extensive human side effect profiles are not well-documented. Based on its mechanism it may cause sedation, dizziness, or mild hypotensive effects, but likely less prominent than less selective antagonists
Pizotifen	Prevent recurrence of migraine and cluster headaches (Fragoso et al., 2021)	~2 nM (Dixon et al., 1977)	Strong antagonist at H_1_ Also an antagonist at 5-HT_1_ receptors and α_1_- and α_2_-adrenergic, dopamine, and muscarinic receptors (Dixon et al., 1977; Przegaliński et al., 1979)	Unclear	22.6 hours (Meier and Schreier, 1976)	Proven effectiveness in attenuating psychedelic experience. Not widely available and large side effect profile including significant sedation and drowsiness, as well as dry mouth, nausea, and dizziness (Fenstermacher et al., 2011)
Ritanserin	Antidepressant, anxiolytic, adjunct in schizophrenia (research use, not widely approved)	0.1–1 nM (very high affinity; Leysen et al., 1985). Inverse agonism in frontal cortex (Muneta-Arrate et al., 2025)	α_1_-adrenergic antagonist, 5-HT_2C_ antagonist, H_1_ antagonist (Leysen et al., 1985)	~1 hour (Zazgornik et al., 1991)	~40 hours (Zazgornik et al., 1991)	Potential anxiolytic effects. Not widely available in United States or Europe for clinical use; primarily a research tool. May cause sedation, orthostatic hypotension, dizziness, dry mouth, weight gain (Akhondzadeh et al., 2008)

Pirenperone has been shown to completely block the psychedelic effects of LSD in preclinical studies (Colpaert et al., 1982; Mokler et al., 1985). It exhibits a short half-life of approximately 4–6 hours, (Janssen Pharmaceutica v. Mylan Pharmaceuticals, 2007) making it another viable candidate. However, pirenperone, despite strong preclinical evidence, has never been approved for clinical use and remains restricted to research settings.

Another selective 5-HT_2A_ antagonist, pimavanserin, has a slower onset (peak concentration around 6 hours) and a prolonged half-life of 57 hours, with its active metabolite (AC-279) exhibiting a half-life of approximately 200 hours (Kitten et al., 2018). Of note, pimavanserin displays different activity at the G_αi1_-protein (inverse agonist) than at G_αiq/11_-protein subunits of the 5-HT_2A_ receptor (Muneta-Arrate et al., 2020, Muneta-Arrate et al., 2025). This leads to different activity in the frontal cortex than at other neurons, and may be of particular importance given that the hallucinogenic-like effects of psychedelics are hypothesized to be mediated through the G_αi1_-protein (López-Giménez and González-Maeso, 2018). Pimavanserin is approved for Parkinson’s disease psychosis; however, it is currently prohibitively expensive, further limiting its utility in acute settings. Other candidate 5-HT_2A_ antagonists, for example, ritanserin (which also displays inverse agonism at the G_αi1_-protein) and piremperone, have been developed or are in the development process (Muneta-Arrate et al., 2025). However, they have significant side effects (see Table 2) or are not widely available, which may prevent clinical trial exploration and potential pragmatic consideration. Pizotifen, an anti-migraine medication, does not display the same selectivity as previously mentioned medication in this class, however it is a potent 5-HT_2A_ antagonist and has been shown to attenuate the effect of MDMA in rats, notably outperforming both clozapine and cyproheptadine in reducing MDMA responses (Young et al., 2005). Moreover, in rodents, pizotifen pre-treatment has been shown to attenuate several psychedelic agents, including, LSD and mescaline (Fiorella et al., 1995; Winter, 1978). Pizotifen is widely available, though mainly outside of the United States and its use is complicated by its side effect profile, which includes antihistaminergic and anticholinergic effects. The trip-killing effects of the medication in humans remain to be tested.

In summary, while selective 5-HT_2A_ antagonists offer a rational approach to terminating psychedelic experiences, their success may depend as much on the binding kinetics of the psychedelic as on the properties of the antagonist itself. Among this class, ketanserin and pirenperone stand out as the most plausible candidates for clinical use as trip killers. Nevertheless, the aforementioned limitations underscore a pressing need for targeted drug development or repurposing, with careful consideration of availability, pharmacokinetics, safety, and regulatory feasibility. For these reasons, we shall next consider potential agents that may be used to attempt to terminate a bad trip in the absence of a specific 5-HT_2A_ antagonist.

### Drugs for psychosis

Table 3 shows relevant characteristics of select antipsychotics. A recent systematic review found second-generation antipsychotics to be broadly effective and tolerable in managing psychedelic-induced persistent psychosis, a far rarer and more severe presentation than bad trips (Sulstarova et al., 2025). Here, we focus on the most pertinent and evidence-based drugs within the class. The limited literature available on the effects of antipsychotics on the psychedelic state suggests that only those with high 5-HT_2A_ activity will reliably attenuate its effect; others may instead operate in other avenues, such as via sedation. Second-generation antipsychotics are typically classified by their antagonism of the 5-HT_2A_ receptor, while first-generation antipsychotics such as haloperidol have low to no affinity at this site, with the notable exception of chlorpromazine.

**Table 3.** Pharmacological properties for select drugs for psychosis: All act as inverse agonists at 5-HT_2A_ unless otherwise specified.

Drug	Primary use	5-HT_2A_ binding affinity (Ki)	Additional receptor profile	Time to peak plasma level	Half-life	Advantages/disadvantages
Aripiprazole	Schizophrenia, bipolar disorder, major depressive disorder (adjunct), Tourette’s	3–25 nM (high/moderate affinity) partial agonist (Kroeze et al., 2003; Sparshatt et al., 2010)	D_2_ partial agonist 5-HT_1A_ partial agonist, α_1_-adrenergic antagonist, H_1_ antagonist (Kroeze et al., 2003)	3–5 hours (Sparshatt et al., 2010)	75–94 hours (parent drug; Sparshatt et al., 2010)	More favorable side effect profile disintegrating tablet, and IM forms. Long half-life, akathisia, nausea, vomiting, constipation, insomnia, restlessness (Sparshatt et al., 2010)
Asenapine	Schizophrenia, bipolar I disorder (mania and mixed episodes)	0.1–1 nM (very high affinity; Shahid et al., 2009)	High affinity antagonist at D_1,2,3,4_, and 5-HT_2B_, 5-HT_2C_, α_1_, H_1_, and muscarinic receptors (Shahid et al., 2009)	0.5–1 hours (sublingual; Bartlett and Van Der Voort Maarschalk, 2012)	24 hours (Bartlett and Van Der Voort Maarschalk, 2012; Citrome, 2014)	Low EPS risk; rapid onset of action (sublingual). Must be taken sublingually, significant sedation. Oral hypoesthesia, somnolence, dizziness, oral hypoesthesia (Gonzalez et al., 2011)
Brexpiprazole	Schizophrenia, major depressive disorder (adjunct), agitation associated with dementia due to Alzheimer’s disease	0.5–2 nM (high affinity) partial agonist (Stahl, 2016)	D_2_ partial agonist, 5-HT_1A_ partial agonist, 5-T1_B_/α_2C_ adrenergic antagonist. (CHECK; Stahl, 2016)	4 hours (Das et al., 2016)	91 hours (parent drug; (Das et al., 2016)	High affinity antagonist at 5-HT_2A_, and a partial agonist at 5-HT_1A_. Long half-life and time to peak plasma level; higher cost. Akathisia, dizziness, headache, nasopharyngitis (Stahl, 2016)
Chlorpromazine	Schizophrenia, bipolar mania, severe behavioral problems in children, intractable hiccups, nausea/vomiting, pre-operative sedation	25–75 nM (moderate affinity) (in vitro) metabolites lack 5-HT_2A_ activity (Kroeze et al., 2003, Suzuki et al., 2013)	D_1_, D_2_, D_3_, D_4_, H_1_, M_1_, α_1_-adrenergic antagonist (Kroeze et al., 2003, DeVane, 1995)	2–4 hours (DeVane, 1995)	10–30 hours (DeVane, 1995)	Research showing a lack of effect when co-administered with LSD (Isbell and Logan, 1957). High risk of extrapyramidal side effects significant sedation; orthostatic hypotension; anticholinergic side effects
Loxapine	Schizophrenia, acute agitation associated with schizophrenia or bipolar I disorder	2–8 nM (Ferreri et al., 2018; Kroeze et al., 2003)	Antagonist at D_1_, D_2_, D_3_, and D_4_, 5-HT_2C_, α_1_ adrenergic receptor; and H_1_ (Kroeze et al., 2003)	1 hour (oral). 2–3 minutes (inhaled; Popovic et al., 2015)	4 hours (Popovic et al., 2015)	Rapid-acting inhaled formulation. Not widely available. Relatively high propensity for EPSEs, as well as sedation and orthostatic hypotension (Popovic et al., 2015)
Lurasidone	Schizophrenia, bipolar depression	2 nM (high affinity; Ishibashi et al., 2010)	High affinity antagonist for D_2_, 5-HT_7_, α_2C_ (Ishibashi et al., 2010)	1–3 hours (with food; Greenberg and Citrome, 2017)	18 hours (Greenberg and Citrome, 2017)	Lower metabolic effects. Must be taken with food (at least 350 calories) for adequate absorption. Akathisia, somnolence, nausea, Parkinsonism, agitation, weight gain (low; Jaeschke et al., 2016)
Olanzapine	Schizophrenia, bipolar I disorder, agitation associated with schizophrenia or bipolar I mania	4–30 nM (high affinity; Kroeze et al., 2003; Ishibashi et al., 2010)	D_1_, D_2_, D_4_, 5-HT_2C_, H_1_, M_1–5_ (anticholinergic), α_1_-adrenergic antagonist (Kroeze et al., 2003; Ishibashi et al., 2010)	5–8 hours (Callaghan et al., 1999)	21–54 hours (Callaghan et al., 1999)	Widely available and used for behavioral disturbance, available in IM form relatively low EPS risk. High risk of sedation; moderate anticholinergic effects. Sedation, weight gain, orthostatic hypotension, dry mouth, constipation, dizziness, akathisia (less common than typicals; Conley and Meltzer, 2000; Leucht et al., 1999)
Paliperidone	Schizophrenia, bipolar I disorder (mania and mixed episodes), irritability associated with autistic disorder	~1 nM (Kroeze et al., 2003; Schotte et al., 1996)	High affinity for D_2_; also α_1_-adrenergic and H_1_ antagonism (Kroeze et al., 2003; Ishibashi et al., 2010)	1–2 hours (for risperidone). 3–4 hours (for active metabolite, paliperidone; De Leon et al., 2010)	20–25 hours (De Leon et al., 2010)	High binding affinity for 5-HT_2A_. Higher risk of EPS and prolactin elevation (compared to other atypicals), orthostatic hypotension. Sedation, extrapyramidal side effects (e.g., akathisia, Parkinsonism), orthostatic hypotension. (Hodkinson et al., 2021)
Quetiapine	Schizophrenia, bipolar disorder (mania, depression, maintenance), major depressive disorder (adjunct)	14–80 nM (moderate affinity; Kroeze et al., 2003; Nasrallah, 2008; Richelson and Souder, 2000)	High affinity for H_1_; moderate affinity for α_1_-adrenergic; lower affinity for D_2_, M_1_. Its active metabolite, norquetiapine, has notable effects on noradrenaline transporter inhibition (Richelson and Souder, 2000)	1.5–2 hours (Han et al., 2024)	6–7 hours (Han et al., 2024)	Low EPS risk; shorter half-life. Significant sedation (especially at lower doses); orthostatic hypotension. Sedation, dizziness, orthostatic hypotension, dry mouth, weight gain, constipation, elevated liver enzymes (Leucht et al., 1999)
Risperidone	Schizophrenia, bipolar I disorder (mania and mixed episodes), irritability associated with autistic disorder	0.1–1 nM (Kroeze et al., 2003; Schotte et al., 1996)	High affinity for D_2_; also α_1_-adrenergic and H_1_ antagonism (Kroeze et al., 2003)	1–2 hours (De Leon et al., 2010)	3–20 hours (De Leon et al., 2010)	High binding affinity for 5-HT_2A_. Available in IM form (would allow for more rapid absorption still). Higher risk of EPS and prolactin elevation (compared to other atypicals), orthostatic hypotension. Sedation, extrapyramidal side effects (e.g., akathisia, Parkinsonism), orthostatic hypotension. (Hodkinson et al., 2021)
Ziprasidone	Schizophrenia, acute treatment of manic or mixed episodes in bipolar disorder	0.1–1 nM (Kroeze et al., 2003; Kongsamut et al., 2002 **)**	Potent 5-HT_1D_ receptor antagonist and a 5-HT_1A_ partial agonist, blocks serotonin and noradrenaline transporters(Ablordeppey et al., 2008; Kroeze et al., 2003)	6–8 hours for oral (Stip et al., 2011). 1 hour for IM	4–10 hours (Stip et al., 2011)	Pharmacokinetics make its oral use infeasible. Long time to peak plasma and its bioavailability of oral ziprasidone is dependent on administration with food (reduces by half from 60% if taken in a fasted state (Mattei et al., 2011). The bioavailability of the IM preparation is closer to 100%

Analysis of online forum discussion of bad trips, albeit not specific to psychedelics, identified antipsychotics as a common method to mitigate negative experiences (Deluca et al., 2012; Valeriani et al., 2015). Olanzapine, quetiapine, and risperidone were the three most discussed medications used, with olanzapine frequently identified as the trip killer of choice. While these reports suggest perceived utility, they are limited by anecdotal biases and lack of clinical oversight. Although many antipsychotics, for example, risperidone, olanzapine, and clozapine, may act as inverse agonists at the 5-HT_2A_ receptor, they have different functional selectivity for the receptor subtypes and activation of beta arrestins (Gaitonde et al., 2024); with risperidone operating with the greatest potency across different 5-HT_2A_ receptor sub-types. Other antipsychotics, that is, aripiprazole and haloperidol have specific areas of non-activity at the G-Protein Couple Receptor (GPCR) sites and beta arrestins, which could limit their ability to attenuate the effects of psychedelics on the 5-HT_2A_ receptor.

Vollenweider et al. (1998) showed that the psychedelic effects of psilocybin were blocked dose-dependently by pre-treatment with risperidone, with 1 mg blocking 98% of the effects. When haloperidol, a potent dopamine antagonist with limited serotonergic binding, was administered before psilocybin, participants experienced fewer feelings of boundlessness, and less derealization and depersonalization phenomena, but reported increased anxiety without any effect on the prominence of visual hallucinations. This finding suggests certain antipsychotics may not only be ineffective but may also be detrimental when administered during a bad trip. Given that haloperidol is frequently part of rapid tranquilization protocol, this lack of efficacy in attenuating and possibly exacerbating the psychedelic state should strongly be borne in mind. Furthermore, the limited literature available on the effects of antipsychotics on the psychedelic state suggests that only those with 5-HT_2A_ activity reliably attenuate its effect (Halman et al., 2024).

Olanzapine and clozapine are two important potential antipsychotic agents that could be considered in the treatment of a bad trip and are widely available. Both agents act as antagonists with high binding affinity at the 5-HT_2A_ receptor (see Table 3). However, the side effect profile and long half-life of olanzapine may prevent its clinical usage as individuals are likely to be sedated for a long period following drug administration. Empirical evidence for olanzapine’s effect in attenuating psychedelic experiences is limited, although an RCT is currently being run to compare its effects to ketanserin and lorazepam (U.S. National Library of Medicine, 2023). Although clozapine has high affinity to the 5-HT_2A_ receptor, its risk and side effect, including agranulocytosis, would make the drug untenable as an option. Chlorpromazine, despite being termed a first-generation or typical antipsychotic, has a higher affinity for the 5-HT_2A_ receptor than it does for any dopamine receptor, although its metabolites mean this effect is reduced in vivo (DeVane, 1995; Suzuki et al., 2013). Early literature investigated the effects of oral chlorpromazine administration on LSD, finding that doses of 75 mg did not attenuate the effects of LSD when given concomitantly to a small sample of males with addiction issues (Isbell and Logan, 1957). The use of chlorpromazine has widely fallen out of practice due to its large side effect profile which, in the case of once-off administration, would include sedation, hypotension, and dizziness.

Ziprasidone, lurasidone, loxapine, and asenapine are four potential candidate treatments for bad trips, all of which have high affinity for the 5-HT_2A_ receptor (see Table 3 for comparison), likely capable of displacing LSD. Ziprasidone can be given orally or intramuscularly and has a favorable peak serum concentration (1 hour) when administered intramuscularly and 6–8 hours orally, with a half-life of 10 hours. Ziprasidone’s oral bioavailability roughly doubles when taken with food, (Miceli et al., 2007) and is sedating in nature. Nevertheless, the side effect profile of ziprasidone is relatively positive. Lurasidone acts as a partial agonist at 5-HT_2A_ receptor and must be taken with food, which could limit its use in managing bad trips. Psychedelics are often consumed on an empty stomach, and it may be difficult to entice a person in extreme distress to consume the required food. Both lurasidone and ziprasidone have strong sedating effects. Loxapine has moderately high affinity for both the 5-HT_2A_ and D_2_ receptors, and can be administered orally, intramuscularly, or via inhalation (Popovic et al., 2015). Asenapine, although lacking in direct evidence, appears to be a compelling candidate. It combines potent 5-HT_2A_ receptor antagonism with rapid absorption through its sublingual administration. This sublingual administration and the need for the person to not eat or drink for ten minutes after administration (Bartlett and Van Der Voort Maarschalk, 2012) may prove challenging, though not insurmountable, to a person experiencing a bad trip. Additionally, the longer half-life of asenapine (24 hours) may be a deterrent.

Other notable candidates among this class are aripiprazole and brexpiprazole, both of which act as antagonists at 5-HT_2A_ and as partial agonists at 5-HT_1A_ receptor [both pre- and post-synaptically; (Stahl, 2021)]. Given the potential for 5-HT_1A_ activity to modulate the 5-HT_2A_ receptor, as seen with buspirone, further investigation with aripiprazole and brexpiprazole as potential trip killers is warranted. Nevertheless, the pharmacokinetics of both are unfavorable in managing a bad trip, as both have long half-lives and times to peak plasma. This would be less problematic if aripiprazole were given in its intramuscular form, which would allow for more rapid absorption. Other antipsychotic medications, such as zotepine and sertindole, may be limited due to their lack of approval in many countries globally, although both may have potential due to their high 5-HT_2A_ receptor antagonism.

Interestingly, among antipsychotics, quetiapine was reported by recreational users in online forums to be by far the most commonly self-administered trip killer specifically for psychedelics (Yates and Melon, 2024), despite its lower affinity for the 5-HT_2A_ receptor compared to the antipsychotics mentioned thus far (see Table 3). Quetiapine displays far higher affinity for the histamine-1 (H_1_) receptor than the 5-HT_2A_ receptor, meaning lower doses would result in sedation without treating a psychedelic crisis, which would require more moderate doses. Quetiapine’s relatively lower D_2_ receptor blockade would reduce the risk of extrapyramidal symptoms, and its short half-life of 6 hours would minimize lasting effects. However, its weaker antagonism at 5-HT_2A_ receptors and its longer peak plasma time (90 minutes) compared to risperidone, means its use may be more beneficial in those for whom sedation would be beneficial, or those who are already taking a selective antidopaminergic agent.

A major advantage of antipsychotics is that they are far more available and affordable compared to selective 5-HT_2A_ receptor antagonists at this timepoint. As such, they should be accessible to clinicians, patients, and researchers, while also being available in a variety of forms of administration, for example, orally, intramuscular, and intravenous. There is also a high degree of confidence and knowledge with this class of medication among clinicians in both psychiatry and emergency medicine, which may more naturally lead to more uptake and appropriate treatment. Antipsychotics also have the obvious benefit of treating an acute psychotic episode in the absence of information about recent psychedelic administration. Among antipsychotics, risperidone may be recommended as the first-line treatment for a bad trip, based on the limited evidence in humans to date as well as its safety and receptor profile. Paliperidone, the active metabolite of risperidone, may be considered as an alternative, as it bypasses CYP2D6 metabolism, which can be problematic in slow metabolizers. Other potentially suitable options, in approximate order of risk-benefit, include asenapine, intramuscular ziprasidone, loxapine, and lurasidone.

### Drugs for depression and anxiety

Table 4 displays the relevant characteristics of select antidepressants. Antidepressants with 5-HT_2A_ antagonistic properties, such as mirtazapine and trazodone, provide a theoretical basis for mitigating negative psychedelic experiences by blocking the primary receptor targeted by classic psychedelics. Anecdotal reports from online sources suggest trazodone is the most used antidepressant by recreational users seeking to terminate a bad trip (Yates and Melon, 2024). Supporting this, a single case report and a 24-week pre-treatment study demonstrated that trazodone (200 mg total daily dosage) attenuated the psychological and physiological effects of LSD (Bonson et al., 1995). Trazodone has moderate affinity for the 5-HT_2A_ receptor (Cusack et al., 1994; Rothman et al., 2000). Trazodone’s time to peak plasma of 1 hour would suggest it could be suitable in acutely treating bad trips, and its shorter half-life of 5–9 hours may allow for more flexible dosing in acute settings (Kale and Agrawal, 2015). Mirtazapine has a moderate affinity for the 5-HT_2A_ receptor and a longer half-life, which may preclude its usage (Fernández et al., 2005; Kennis et al., 2000). Both medications are sedating and have a strong affinity for the histamine 1 receptor, which may simply sedate the person rather than terminate the trip.

**Table 4.** Pharmacological properties of select antidepressants/anxiolytics.

Drug	Primary use	5-HT_2A_ binding affinity (Ki)	Additional receptor profile	Time to peak plasma level	Half-life	Advantages/disadvantages
Buspirone	Generalized anxiety disorder	Partial agonist, ~100–300 nM (low direct affinity for 5-HT_2A_ (Bronowska et al., 2001; Hjorth and Carlsson, 1982)	Full agonist at presynaptic 5-HT_1A_ autoreceptors. Partial agonist at postsynaptic 5-HT_1A_. Weak antagonist at D_2_ and α_1_-adrenergic receptors (Bronowska et al., 2001)	0.667–1.5 (40–90 minutes; Gammans et al., 1986)	2–3 hours (Gammans et al., 1986)	Non-sedating; has shown an effect in attenuating the effects of psychedelics. (Pokorny et al., 2016). Short half-life (could be overcome with repeated dosing). Dizziness, nausea, headache (O’Hanlon, 1991)
Mianserin	Major depressive disorder, anxiety disorders (particularly where sedation is desired)	4.3–10 nM (high affinity; Mylecharane, 1991)	α_2_-adrenergic autoreceptor antagonist, strong H_1_ inverse agonist, 5-HT_1D_, 5−HT_2B_, 5−HT_2C_, 5-HT_3_ antagonist. Weak norepinephrine reuptake inhibitor (Mylecharane, 1991)	1–3 hours (oral; Shami et al., 1983)	6–39 hours (Shami et al., 1983)	Limited advantages. Risk of blood dyscrasias (e.g., agranulocytosis) outweighs any benefit. Significant sedation, orthostatic hypotension (Clink, 1983)
Mirtazapine	Major depressive disorder, anxiety disorders, insomnia	8–70 nM (moderate affinity; Fernández et al., 2005, Kennis et al., 2000)	α_2_ adrenergic antagonist (presynaptic, increasing NE, 5−HT_2C_ antagonist, H1 antagonist, 5-HT_3_ antagonist (Millan et al., 2000)	2 hours (Timmer et al., 2000)	20–40 hours (Timmer et al., 2000)	Widely used in a variety of medical settings. Significant sedation. Sedation, increased appetite, dizziness, dry mouth, constipation
Trazodone	Major depressive disorder, anxiety disorders, insomnia	~20 nM (Cusack et al., 1994, Rothman et al., 2000)	α_1_-adrenergic antagonist, H_1_ antagonist, weak 5-HT reuptake inhibitor (Mandrioli et al., 2018)	0.5–2 hours (Kale and Agrawal, 2015)	5–9 hours (Kale and Agrawal, 2015)	Rapid onset of action and short half-life. Significant sedation; orthostatic hypotension; risk of priapism (rare but serious). Sedation, dizziness, orthostatic hypotension, dry mouth, nausea, blurred vision, priapism (rare)

Selective serotonin reuptake inhibitors are the most prescribed class of antidepressants and have displayed mixed results regarding their effect on dampening a psychedelic experience (Becker et al., 2022; Gukasyan et al., 2023). Studies investigating concomitant SSRI and psychedelic use have involved participants who had prolonged SSRI use, rather than a once-off dose (Goodwin et al., 2023). A recent preclinical study indicates that an increase in serotonin at the synaptic cleft may diminish the psychedelic response, with the authors speculating that the increased serotonergic tone could lead to indirect functional antagonism of psychedelic effects by causing an imbalance of 5HT_1a_ and 5HT_2a_ receptor activation (Erkizia-Santamaría et al., 2025). Although this represents a promising avenue for human research, SSRIs are a priori unlikely to match the trip-killer potency of the previously discussed medications. Tricyclic antidepressants such as amitriptyline, clomipramine, and imipramine generally combine, among other actions, antagonism of the 5-HT_2A_ receptor with blockade of the serotonin (SERT) and norepinephrine reuptake transporter (NERT; Danish University Antidepressant Group, 1999; Lawson, 2017). Indeed, chronic use of tricyclics such as clomipramine and imipramine was shown to increase the psychological effects of LSD (Bonson and Murphy, 1995). Other antidepressants, such as mianserin and nefazodone, could be alternative candidates; however, they suffer from significant risk profiles. Thus, among antidepressants, trazodone and mirtazapine appear to be the more favorable candidates, and with the former perhaps an adjunctive or secondary option when antipsychotics are contraindicated or unavailable.

### Antihistamines

Table 5 shows the relevant characteristics of select antihistamines. Some antihistamines, such as cyproheptadine, combine strong histamine-1 (H_1_) receptor antagonism (Ki 1–2 nM) with notable 5-HT_2A_ antagonism (Ki = 20 nM), which positions them as intriguing candidates for counteracting psychedelic effects (Honda et al., 2003). Cyproheptadine’s half-life of about 8 hours allows for a moderate duration of action; it also has a peak plasma concentration of 1–3 hours. Preclinical reports indicate that it may blunt psychedelic-induced neuronal activity in cats (Wilkison and Hosko, 1983) but human evidence is lacking, and its sedative and anticholinergic burden raise concerns. Alternatively, promethazine offers a similar pharmacodynamic and 5-HT_2A_ receptor binding profile to cyproheptadine but with a longer half-life. Overall, while antihistamines lack the targeted 5-HT_2A_ antagonism of certain antipsychotics or antidepressants, their sedative (e.g., diphenhydramine) and anxiolytic effects may provide symptomatic relief during difficult psychedelic episodes. Their widespread availability and favorable safety profiles make them pragmatic options when more specific agents are unavailable. Nonetheless, their limited receptor specificity means they are unlikely to terminate the core psychedelic experience rapidly. Future research should clarify their role as adjunctive agents in the management of acute psychedelic distress.

**Table 5.** Pharmacological properties of select antihistamines.

Drug	Primary use	5-HT_2A_ binding affinity (Ki)	Additional receptor profile	Time to peak plasma level	Half-life	Advantages/disadvantages
Cyproheptadine	Atopic conditions, management of serotonin syndrome, migraine prophylaxis	1–2 nM (high affinity; Honda et al., 2003)	Potent antagonist at H_1_ and 5-HT_2C_. M1 antagonist, α_1_-adrenergic antagonist (Honda et al., 2003)	1–2 hours (Gunja et al., 2004)	8.6 hours (Gunja et al., 2004)	Short peak plasma time and half-life. Significant sedation and anticholinergic effects; sedation, dry mouth, dizziness, constipation, blurred vision, weight gain
Diphenhydramine	Allergic symptoms, insomnia, motion sickness	Low affinity (Nerush et al., 2024)	Strong H_1_ and M_1_ antagonist. α_1_-adrenergic antagonist, weak Na+ channel blocker (Ghoneim et al., 2006)	1–2.7 hours (Electronic Medicines Compendium, 2025; Scavone et al., 1998)	2–9 hours ( Electronic Medicines Compendium, 2025; Scavone et al., 1998)	Widely available over the counter. Low affinity for 5-HT_2A_; sedative and anticholinergic side effects. Sedation, dry mouth, blurred vision, urinary retention, constipation
Promethazine	Allergic conditions, nausea/vomiting, insomnia, pre-operative sedation	Low affinity (DrugBank, 2025)	Potent H_1_ antagonist, D_2_ antagonist (weak), M_1_ antagonist (strong), α_1_-adrenergic antagonist (DrugBank, 2025)	2–4 hours (Church and Church, 2013)	10–19 hours (Church and Church, 2013; Paton and Webster, 1985)	Anti-emetic effect. Available in IM form Sedation; anticholinergic side effects; can cause EPS (especially in children/elderly). Sedation, dry mouth, blurred vision, constipation, dizziness, urinary retention, EPS (rare)

### Benzodiazepines

Benzodiazepines appear to be widely used to manage acute distress during challenging psychedelic experiences (Johnson et al., 2008). Online forum analyses revealed that 46% of posts on Reddit regarding trip killers referenced use of this medication class, with alprazolam and diazepam being the most prevalent compounds discussed (Yates and Melon, 2024). Unlike the agents listed above, benzodiazepines do not directly affect the serotonergic system. As such, they would not terminate the psychedelic experience itself but would instead provide potent anxiolysis or sedation. Indeed, based on their experiences in administering psilocybin, Johnson et al. (2008) propose 10 mg of oral diazepam as a sufficient dose to aid anxiolysis in the context of a bad trip; they add that the rapid onset and high lipid profile are particularly useful characteristics of the drug. Midazolam is commonly used in medical settings, and its IV and oral formulation achieve peak effect in 5 and 30 minutes, respectively, with a half-life of <2 hours (Nordt and Clark, 1997). Agents such as lorazepam, diazepam, and clonazepam have rapid onsets (minutes to an hour) and half-lives ranging from approximately 12 to 48 hours, providing both immediate and sustained anxiolytic effects during crises (Riss et al., 2008). Benzodiazepines, therefore, offer a promising adjunct to 5-HT_2A_ antagonists or a primary option for those who would prefer anxiolysis while persisting with the psychedelic experience. However, their use would be less appropriate in the non-clinical environment due to their risk of addiction, as well as respiratory depression, hypotension, and reduced levels of consciousness.

### Niacin

One small but notable controlled study demonstrated that a high dose (3 g) of niacin (vitamin B3) exhibited a blocking action on the psychoactive effects of LSD (Agnew and Hoffer, 1955). The study found that pre-treatment with niacin led to a delayed onset of LSD-induced effects and significantly reduced the occurrence of perceptual distortions typically associated with the drug. Remarkably, when niacin was administered after LSD ingestion, it rapidly attenuated all effects of the psychedelic within 5 minutes. Niacin was also commonly used by George Peters’ “LSD Rescue Group” to purportedly remarkable effect in terminating bad trips, (Peters, 1970) although objective evidence is lacking and the mechanisms underlying this effect remain speculative. Niacin is not known to act as a direct serotonin receptor antagonist, although has been observed to release serotonin in platelets (Papaliodis et al., 2008). Niacin activates peripheral prostaglandin-mediated vasodilation, through GPR109A receptors, causing a dermatological flushing and a burning sensation on the skin. In bad trip contexts its main effect was possibly to shift attention to bodily discomfort. Modern replication studies would appear to be warranted before niacin could be considered a plausible trip killer.

### Unhelpful strategies

Alcohol and cannabis may be used by individuals in an unsupervised setting, (Lake and Lucas, 2025; Yates and Melon, 2024) but are more likely to be harmful than helpful. Alcohol’s impairment of cognitive and motor function can increase the risk of accidents or risky behavior during a psychedelic experience. Combining alcohol with psychedelics can also increase the likelihood of nausea and vomiting due to additive gastrointestinal irritation (Johnson et al., 2008). Cannabis may intensify visual distortions, emotional lability, and anxiety during a psychedelic trip despite being commonly used to reduce post-acute psychedelic anxiety or to ease the transition back to baseline consciousness (Johnson et al., 2008). The precise impact of cannabis on the psychedelic experience may depend on timing, dose, and individual neurobiology, but its potential to enhance or complicate the psychedelic experience warrants caution.

## Future research

Much of the current understanding of pharmacological trip termination remains theoretical, with limited direct experimental evidence in humans. Agents such as ketanserin have demonstrated the central role of 5-HT_2A_ receptor antagonism in modulating psychedelic experiences, yet systematic research on managing distressing psychedelic states, reducing intensity, shortening duration, or fully terminating effects is sparse. Ethical constraints preclude studies designed to deliberately induce bad trips, but investigating medications’ impact at peak effect may provide a useful proxy (U.S. National Library of Medicine, 2025). However, future clinical trials could incorporate abortive strategies prospectively. For example, participants could be given the option to request administration of a 5-HT_2A_ receptor antagonist such as ketanserin if acute distress arises. This would enable systematic assessment of efficacy, safety, and tolerability in terminating or mitigating negative experiences without compromising therapeutic outcomes. Comparative studies of different trip-killing agents with diverse receptor profiles are needed to optimize treatment protocols, and a trial has been completed to assess the impact of ketanserin, olanzapine, lorazepam, or placebo post-consumption of LSD. Beyond pharmacodynamics, future research must explore patient and clinician preferences, ethical considerations, and the impact on the overall psychedelic therapeutic process. Additionally, the frequent co-use of other substances during psychedelic experiences warrants investigation into poly-substance interactions and their neuropharmacological implications. Lastly, 5-HT_2A_ receptor agonists such as lisuride and ergotamines do not produce psychedelic effects, which suggests that 5-HT_2A_ receptor agonism is not causatively sufficient for a psychedelic experience (Adams and Geyer, 1985, Marona-Lewicka et al., 2002). Additionally, psychedelics such as 4-bromo-2,5-dimethoxyphenethylamine (2-CB) act as low-efficacy partial agonists/functional antagonists, yet produce strong psychedelic effects (Marcher-Rørsted et al., 2020). This may be related to the fact that each psychedelic has a complex and unique pharmacological profile at the 5-HT_2A_ receptor, binding to distinct locations within the G-Protein Coupled Receptor (GPCR). As such, the most effective trip killer may need to have effects beyond simple 5-HT_2A_ receptor antagonism. Addressing these gaps through rigorously designed, ethically sound clinical studies will be critical for developing evidence-based interventions to manage psychedelic crises effectively.

## Recommendations

We preface these recommendations by acknowledging that they are based mostly on theory, rather than empirical evidence. Nonetheless, they may offer guidance to researchers or clinicians dealing with an individual in the throes of a bad trip for whom non-pharmacological methods have been exhausted or are inappropriate. Any individual to whom medication is administered should be frequently observed for the side effects outlined in Tables 2–4.

Given its strong theoretical basis and direct experimental evidence, ketanserin may be proposed as a first-line agent in managing a bad trip. If ketanserin is unavailable, risperidone (or the primary active metabolite, paliperidone) is an appropriate alternative. Risperidone’s widespread availability in oral and intramuscular forms makes it more feasible to use than two other strong trip-killing candidates: lurasidone, which would require concomitant food ingestion, or asenapine, which requires sublingual administration, both of which could be difficult to administer to a person in the extreme distress necessitating pharmacological treatment. Should the individual have a history of neuroleptic sensitivity, cyproheptadine, trazodone, or mirtazapine are proposed alternatives. The sedation caused by these medications may be sought after, but they lack the strong 5-HT_2A_ antagonism of the previously listed medications.

## Conclusion

The increasing prevalence of unsupervised psychedelic use presents significant challenges for healthcare providers, particularly in acute care settings that are not equipped to support altered states of consciousness. Psychological containment and non-pharmacological interventions should be prioritized to preserve the therapeutic potential of difficult psychedelic experiences. However, there remains a clinical need for careful pharmacological intervention when safety risks arise. Medications that block the 5-HT_2A_ receptor, such as ketanserin, pimavanserin, or atypical antipsychotics including risperidone, asenapine, and lurasidone, may offer effective options. Alternative agents like olanzapine, trazodone, or quetiapine could also be considered, with benzodiazepines serving as possible adjuncts. These medications should be used with caution to enhance safety while avoiding the premature interruption of important emotional processing and insight. As psychedelic use becomes more widespread, healthcare systems will need to adapt to address the associated needs, ensuring patient safety and supporting therapeutic outcomes.
